# Tagebuch einer Medininischen Reise nach England, Holland, und Belgien, von Dr. G. Varrentrapp. (Journal of a Medical Tour in England, &c.) London Hospitals

**Published:** 1840

**Authors:** 


### TAGEBUCH EINER MEDININISCHEN REISE NACH ENGLAND, HOLLAND, UND BELGIEN VON DR. *G. Varrentrapp.* Frankfurt, 1839.

### JOURNAL OF A MEDICAL TOUR IN ENGLAND, &c.

WITHOUT preface we shall introduce our pleasant gossiping author to our readers, and allow him, as far as mere extracts will suffice, to tell his own tale. Like all his countrymen, he is often most perplexingly minute, and that too upon matters which at best are trifling, and without interest. But as he seems to have been quite delighted with all he saw and met with on our shores, we must act as the poet bids us do to the friend we have lost,

> " Be his faults and his follies forgot by thee then."

" On the 8th day of April, 1838, at the hour of noon, I left my old father-town of *Frankfurt,* by the coach—*Eilwagen,* i. e. speed-waggon—for *Mentz,* in a cheerful and easy state of mind; my only regret being that I had not a companion with me, to whom I could speak, and unfold my thoughts, as they arose. For five years I had cherished an anxious wish to visit England, and become acquainted, by a personal interview, with its many wonderful institutions. This wish became stronger and stronger, when the period of my intended departure approached, as the red of the morning sky becomes deeper and deeper, when the rising sun approaches the horizon. I felt confident that the journey would not only renovate the health of my body, but that it would also make young again all the spirits and temper of my mind."

From *Mentz* Dr. V. started next morning before day-light, by the steam-boat, for *Cologne.* The worthy doctor here gives us a minute description of the appearance of the morning sky, the cold frosty looks of his fellow-travellers, the bustle on board the vessel, the gradual rising of the sun, the passing of one or two steam-boats up the Rhine—which, in his usual pleasant garrulity he tells us, caused the appearance of double speed—and the ever varying aspect of each bank of the river: he all the while standing alone and absorbed in a reverie on the bows of the vessel!

Dr. *Varrentrapp* no sooner reached *Cologne* than he forthwith—for, like so many of his countrymen, he is quite an enthusiast—hurried to have a peep at its famous cathedral, and thence to the church of St. Peter, where the last work of Rubens, the Crucifixion of the aged Apostle, is to be seen. This celebrated picture had been taken to Paris to adorn the Louvre, during the Imperial dynasty; but it was returned after the peace to the church from which it had been stolen. From *Cologne* he proceeded to *Nymwegen,* which he reached in the evening, just in time to join some travellers at tea. " Here the smoking tea-urn, the round biscuits of excellent quality, and as light as froth, and either buttered or in company with cheese, the pipes and tobacco, and the guests sitting with their hats on—all shewed that we had left Germany far behind us." *

The next town our gossipping author visited was *Utrecht*, where he tells us that he met with the " most beautiful and national specimen of a Hollander," in the person of his host, that he had yet seen. His round blooming face, dark twinkling eyes, his slightly arched nose, ample forehead, rotund figure, and grave but cheerful manners, seem quite to have captivated the Doctor's fancy.

" The dishes too," he adds, " were equally interesting, being truly national and climatic. Sago-soup with cinnamon and wine, comfrey † with nutmeg, and huge pieces of ginger."

At the *Amsterdam* Hospital he learned that Typhus is by no means of frequent occurrence in Holland; by far the most common class of fevers in the country being of the intermittent kind. Enlargements of the liver, and still more frequently of the spleen, are exceedingly common. Cases of genuine scurvy, too, are often to be seen in the wards.

Dr. *Varrentrapp* being, however, much more minute in his descriptions of the building, of the construction of the various apartments, of the beds, their curtains and so forth, than of the maladies of the inmates, we are unfortunately unable to say more of the diseases of the country, or of the character of its physicians.

One of the pieces of information that he gives us is, that the Syphilitic disease is of unusual inveteracy in many of the towns of Holland,—in consequence, he alleges, of the want of any compulsory regulations for the inspection and management of the *Freuden-Mädchen*, or filles-de-joie. So shocking, says he, is the remissness in this respect, that the physician has it not even in his power to prevent such patients from leaving the hospital before they are cured! In *Leyden*, things are still worse; for at the hospital there they will not even admit any patients affected with syphilis, regarding this disease as a punishment from God upon a vicious life!

" Is not such a custom," exclaims our author, " even worse, more barbarous than that which existed in Germany a century ago, of giving all the venereal patients a good cudgelling on their admission into and their discharge from the hospital!!"

From *Amsterdam*, Dr. V. journeyed to *Haarlem*, and thence to *Leyden*. The university of this latter place still maintains its high repute in Holland: there are usually from 150 to 180 medical pupils studying there. The Museum of Natural History is of surpassing excellence; it is especially rich in all the departments of osteology, human and comparative. The celebrated Professor *Temminck* is the conservator. The *Hague* was the last place he visited before he reached Rotterdam. We find the following medical memorandum of his visit to its Syphilitic Hospital: " The most common

* It is a little curious that the Dutch have appropriated to themselves or rather, perhaps, that we English have given them the proper appellation of the Germans—Deutsch, Deutschland, &c.

† Are we right in our translation of the word *schwartzwurzel*: the same term is applied to the scorzonera, and also to the briony.

method of treatment is the exhibition of *Dzondi's* solution of the corrosive sublimate. Here we saw, God be thanked (Gott sei Dank), a good many cases of the disease in its primary stage. The cause of this is no doubt the circumstance of Dr. *Dingeman*, the physician, having the power of inspecting all the brothels of the place, and of compelling every girl, whom he finds diseased, to go *instanter* into the hospital."

On the whole, Dr. *Varrentrapp* does not seem to have formed a very favourable opinion of the Dutch hospitals or physicians. "The former are usually small, confined, and inconvenient, although clean and tidy; and as to the latter, they may, indeed, be good practitioners, but it must be confessed that certainly their 'outward man' is any thing but prepossessing and professional like. With hat on head, a pen in his right hand, and a cigar almost invariably in his mouth, the Dutch doctor is to be seen, at a stated hour, moving rapidly across the wards of the hospitals, marking down on the card of each patient a prescription, but seldom troubling himself with writing any report of the symptoms." Dr. *Varrentrapp* is very serious in his remonstrances with his Dutch professional brethren on the indecorousness of smoking.

"A physician owes a certain respect to every patient, although he may be the meanest beggar or the greatest rogue: herein consists the beautiful difference between the physician and the lawyer. The scum of mankind come to us medical men, not as criminals who deserve to be punished, even although they may have brought their maladies upon themselves, but as supplicants seeking our aid; and if we can relieve them, they regard us as instruments in the hands of the Almighty. Now this respect must be utterly destroyed by seeing a physician with a cigar in his mouth. A patient may accustom himself to much; but he cannot fail to see that no interest, either scientific or human, no sympathy in his sufferings is felt by a man who is probably re-lighting a cigar, when feeling his pulse, or when looking at his tongue. I do not know whether this practice is ever followed when a physician visits a paying patient."

Whether the custom be the cause or the effect of the imperturbable gravity and love of ease of the Hollander we cannot say; but sure it is, Dr. *Varrentrapp* tells us, that the Dutch doctors of the present day seem to follow their forefathers—and no doubt the forefathers theirs before them—in every respect alike in their mode of life, their mode of dress, and their mode of practice, without minding in the least the noisy changes of this *reforming* age. "He is so enamoured of old habits, that he will admit of no deviation from them. Whatever has existed for a length of time, must be in his opinion right; and all suggestions of improvement are at once checked by the reply, that the present system is found to work admirably. For example, between four and 500 hospital patients are often entrusted to the care of a single physician and his assistant; and it is expected that he should inspect and prescribe for every one of these daily. Now, what is the result? No reports of any of the cases are drawn up; all that is done is the merely writing in a book a certain short prescription opposite to the name of the patient and the number of his bed."

It is a pity that our worthy author, in his zeal to excite a reforming spirit among his Dutch brethren, does not enter a little more into details respecting the medical practice which is pursued by them, and give his readers some idea of their mode of treatment, especially if there be any peculiarity in it, in some class of diseases.

But, as we have already remarked, his attention seems to have been altogether taken up with the construction and arrangement of the buildings, the shape of the beds, the size of the wards, and so forth.

From the *Hague* he travelled to *Delft*—where he visited the tombs of Hugo Grotius and William of Orange, who was so inhumanly murdered—and thence to *Rotterdam*.

As usual, he details all the particulars of his journey—how, for example, the steamer, that brought him over, was obliged by stress of weather to put back to *Helvoetsluys*, where it anchored for the night. "It was very unpleasant thus to spend hour after hour in a most tedious and useless manner, the vessel all the while round-about-tossed—a *Hamiltonian translation*—by the restless waves."

Next morning, however, it crossed the Channel, and the worthy doctor saw for the first time the shores of hospitable England. The voyage up the river interested him, as a matter of course; and most minutely does he describe his astonishment at the number of steam-vessels, small boats, and ships, the buildings on each side, the docks with their forests of masts, the Custom-house, &c. &c.

At length he reaches London, "the *haupstadt* of Europe, that mighty, miles-long central port of the whole world."

After encountering the annoyance of porters, watermen and coachmen, he at length reaches the George and Vulture Tavern at Shadwell, from which, however, he speedily makes a retreat, in consequence of the smoke and darkness of his rooms.* Forthwith he hires a cab, and, impatient of immediately seeing something of the great capital, drives along Leadenhall Street, Cheapside, Holborn, Oxford Street, down Regent Street, and back to the City by the Strand and Fleet Street. "The multitude of coaches, cabs, omnibuses, carts, horses, not to mention the overwhelming number of foot-passengers moving in both directions along the *trottoirs*, is truly astonishing, nay almost frightful to a stranger, and can be compared only to an ant-hill in a state of commotion."

He alludes to the construction of the street cabs—with the driver's seat either above, or before, or behind, or at the side, but always distinct from the body of the vehicle—as very significant of the national character: "an Englishman is far too exclusive to allow a coachman to sit beside him." He then gives a minute description of the tax on tradesmen's carts, which were altogether foreign to him; but our readers will no doubt readily dispense with the particulars.

After the rapid view he thus took of some of the leading streets of the metropolis, our author went systematically to work, and examined all the chief buildings, institutions, manufactories and other *lions* visited by strangers.

As a matter of course he visited Westminster and Henry VII.th's Chapel "with its marvellous treasure of gothic stone-work;" St. Paul's, from whose

* We must not omit to mention that he afterwards found *sehr comfortables*—(this word seems to be naturalised in almost every European language, now-a-days,)—lodgings in Leicester Square.

top the spectator is astonished with "un endliche colossale chaos" of buildings; the Tower, "with its little town within;" the Docks, "those enormous witnesses of the mighty enterprize-spirit of this metropolis of the world's trade;" the Tunnel; the Squares, "the keeping of which closed shews how much the Englander dislikes being intruded on;" the Palaces; the Parks;* the Theatres; the Post Office and its mail-coaches, "which few princely equipages on the Continent equal;" and the numerous Galleries of Painting, not to mention a host of other places.

The worthy doctor seems to have been charmed with the kind reception which he met with every where, and talks in most flattering terms of the inhabitants of "this mighty blooming noble island-land."

"Not only do we perceive at each step the results of individual energy, of a restless activity, of a clear and comprehensive sagacity of every one in his own sphere, of unobstructed and admirably regulated corporation-rights, and of an immoveably secured and a most intimately interwoven freedom; but we see also, on turning our attention to the private and domestic life of the people from that of their business and commerce, a not less beautiful and elevating feature in the former, than a rich, brilliant, and astonishing one in the latter!" * * * "Every Englishman, to whom you may happen to be even accidentally introduced, will gladly assist you, to the best of his abilities, in attaining the objects of your journey, although he can seldom devote his time, at least during the day, to your service. It is unnecessary for me to attempt a description of the family manners of this people, the manner of their home enjoyments with their children and their friends, their dinners and suppers, and their comfortable close houses, as all this has been admirably done by Otto, in his Pictures of London."

We confess that we, as readers, should have been much better pleased with any remarks from so cheerful and so intelligent a traveller as Dr. *Varrentrapp* on our social and scientific institutions, than with the prosy architectural and statistical descriptions with which he has loaded his book. But it is of no use to complain; so let us continue to take him as we find him.

Nearly a hundred pages are occupied with a description of the numerous hospitals, asylums, and dispensaries; their number of beds, their funds and expenditure, the names of the medical officers, and so forth. First, he gives a most exact account of the University College and of King's College, their origin, success, present state, &c.; of the College of Physicians, "where, during the last two years, reform has been making great progress;" of the College of Surgeons, "in whose new building a great luxury prevails;" and of the Hunterian Museum, "so admirably kept and so beautifully arranged by Mr. *Clift* and his son-in-law, Mr. *Owen*;" and lastly of the Society of Apothecaries.

The Hospitals next come under his review; but, as usual, our worthy author is more taken up with the buildings themselves, than with the patients within, or their medical attendants.

*St. Bartholomew's* is most minutely described in all its particulars, down to the shape of its windows, the size of the beds, the construction of its water-closets, and the duties and wages of its nurses. The names indeed of

* Again our author alludes to the *separation-lust* of our countrymen in excluding the public from the Zoological Gardens on Sunday.

the surgeons and physicians are given; but nothing is said as to their practice.

*St. Thomas's* fares somewhat better; for we are told that, "in reference to clinical instruction, this is one of the most interesting hospitals in London. *Travers, Tyrrell,* and *Green* have long been celebrated as operators and teachers: the last usually lives at his country-house, and there pursues his studies, but he visits the hospital twice a-week. The medical clinique of Dr. *Williams* is one of the most pains-taking, most complete and least hurried of any in London. The school of this hospital is therefore much esteemed."

*Guy's* is next noticed; but no remarks are made on the surgeons or physicians. The doctor was present at the annual distribution of prizes to the students, when *der alte Sir Henry Halford* was in the chair, and Dr. *Addison* highly eulogised the treasurer, Mr. *Harrison,* "to whom the hospital is so much indebted in every respect."

Upwards of twelve pages are devoted to the London Hospital, and we are told of every person and of everything connected with it, except of the medical attendants and of their practice.

*St. George's* has only four pages, the University College has six, the Middlesex only half a page, and the Westminster only three lines, giving the names of the physicians and surgeons!

There is a little more interest in the following extract from his remarks on the Fever Hospital.

"During the year 1837—8, the number of fever patients admitted was 946; of whom 218 died. Of the fatal cases, death took place in 16 within 12 hours after admission; in 19 within 24 hours; in 24 within 48 hours; and in 15 within three days.

It thus appears that many of the patients were in the last stage of the disease when received into the hospital. The epidemic of this year was distinguished by certain peculiarities;—viz. by the greater frequency of a petechial eruption, and the more than usual tendency to extreme exhaustion and debility. Hence it has been found necessary to use more wine, brandy, and other stimulants this year than on any former one.

The following table exhibits the number of admissions, and the ratio of the mortality in the different months of the year:

| | Admissions. | | Deaths. | | Proportion. |
|---|---|---|---|---|---|
| January .. | 45 | — | 13 | — | 29 per cent. |
| February .. | 62 | — | 20 | — | 32 " |
| March .. | 75 | — | 60 | — | 21 " |
| April .. | 66 | — | 12 | — | 18 " |
| May .. | 71 | — | 11 | — | 15 " |
| June .. .. | 82 | — | 17 | — | 21 " |
| July .. .. | 100 | — | 22 | — | 22 " |
| August .. .. | 102 | — | 12 | — | 12 " |
| September .. | 80 | — | 21 | — | 26 " |
| October.. .. | 96 | — | 20 | — | 21 " |
| November .. | 91 | — | 24 | — | 26 " |
| December .. | 75 | — | 30 | — | 40 " |
| | 946 | | 218 | | Medium 23." |

Dr. *Varrentrapp* seems to have been particularly pleased with the Ophthalmic Hospital in Moorfields. "The surgeons of this institution, Messrs. *Tyrrell, Scott, Macmurdo* and *Dalrymple* were unusually, even for England, courteous and kind to me. What surprised me much was to find that the *oleum jecoris aselli*—cod liver oil—is seemingly quite unknown here as a remedy in scrophulous diseases. The outward application of calomel too, as recommended by *Fricke*, has never been appreciated here, as this invaluable remedy deserves; and the utility of cold lotions to the eyes after operations is certainly not sufficiently understood by English oculists."

In his notice of Bethlehem Hospital, we were amused with the following passage, where he stands forward as an advocate of Mr. *Lawrence*, or rather as the reprover of the Directors.

"We may here mention a trait of religious feeling on the part of Messieurs the Directors of this institution. *William Lawrence* had published a work on the natural history of man, wherein he had expressed an opinion that the various races of men were probably sprung from more than one pair, as stated in the Mosaic record.

This so offended the Directors that notice was soon given to the author that, unless he suppressed the sale of the book, he must resign his situation as surgeon.

*Lawrence* bought up the impression; but, on a subsequent edition making its appearance, the pious directors of Bethlehem Hospital, believing that it was with the author's cognizance, determined to deprive him of his office. A majority, however, of the subscribers refused their assent to this act of tyranny. And who, pray, were the men who tried thus to stifle a purely scientific enquiry, and to insist upon from others an accordance with their own tenets of belief? They were the same directors who had spent in feasting one half of the income of the establishment—who had caused poor lunatic women to be chained to the walls of their cells—who had fettered the limbs of poor Norris with iron rings round his neck, body, and limbs—and who had imprisoned the amiable and intelligent Mathews.

They would have acted better if, instead of opposing the researches of science, they had devoted their attention with greater zeal to the alleviation of the poor inmates."

Dr. *Varrentrapp* condemns, and with justice too, the custom which has far too long prevailed, of closing this and other public lunatic establishments against visitors, even although they be medical men, unless, indeed, they are introduced by one of the Directors. Attention to the disorders of the mind has hitherto been too much neglected in medical studies; and hence the treatment of this most distressing class of maladies has lapsed into the hands of a few exclusives.

He was much pleased with the County Lunatic Asylum at Hanwell, and with the polite attentions of Dr. *Millengen*, the then Medical Director. With the exception of confining the patients in separate cells, instead of having large apartments where several might sleep together, he highly approved of all the arrangements of this extensive hospital, which usually contains about 700 patients. As many as are fit and willing, are kept engaged in some occupation. Thus, among the men, there were 46 employed as labourers, 42 in gardening, 25 in cleaning the wards, 61 in picking horse-hair, and one in writing accounts, &c.

Among the women, 12 were engaged in the kitchen, 24 in washing, 73

in spinning and sewing, 29 in picking wool, 37 in gardening, 27 in cleaning out the rooms, and 54 in picking horse-hair.

In closing his remarks on the London hospitals, Dr. *Varrentrapp* points out what he considers their chief merits and defects. Among the former, he particularly dwells upon their admirable cleanliness, the excellence of the provisions, the attention of the nurses, and last, though not least, of all, the number and purity of the water-closets—for which, by the bye, there seems to be no word in the German language. The leading defects he enumerates, are the insufficient frequency of the physicians' and surgeons' visits, and the want of any central board of organization, such as the *Conseil general d'administration des Hôpitaux, hospices et secours à Paris.* He mentions also the difficulties in the way of admission for patients, unless they are recommended by some subscriber or governor, and the limitation of this to one or two days only during the week. The University College Hospital is an exception indeed to this censure; for there patients are admitted daily. He goes on to observe :

"It is, however, especially remarkable that, with the enormous sums which are appropriated to the use of the various hospitals in this metropolis, there is on the whole so small a number of beds in them. The leading hospitals contain about 3,000 beds, the hospitals for special complaints about 500 more, and the lunatic establishments about 15 or 16,000. What a small proportion is this—with the exception perhaps of the lunatic establishments—to the population, in comparison with other cities. Francfort, with a population of only 55,000 inhabitants, has 500 beds in its six civil hospitals, besides another 100 for epileptic and lunatic patients ; and Paris, with a population of about 800,000 has 5,000 beds in its hospitals, and 10,000 in its hospices."

In reference to the character of the clinical instruction communicated to students in the London Hospitals, the following observations of our author, however pedantically expressed, will be read with some portion of interest.

"We do not indeed hear either long complete discourses and tedious Socratic disputations, nor profound system demonstrations and curious disquisitions, as in many of the best German clinics ; but we hear something which is perhaps much more incomplete, and far less intellectual, but which nevertheless is much more true and practical.

It is in that national tendency of the English to attend almost exclusively to merely practical subjects, that we are to seek for the cause of the chief differences between the English and the German physicians. We are not to expect to find among the former men of deep and comprehensive acquirements, acquainted with the history of medicine in all languages, such as is the character of some of my countrymen. A careful observation of individual cases, and the comparing of these with similar ones, either in their own practice or in that of others, and withal a practical but not a far-stretching reasoning, directed more especially to therapeutics, seem to constitute the chief ambition and certainly the leading excellence of the English physicians. They have accordingly produced some admirable monographs ; but they seldom trouble themselves about systematic works. The higher fields of speculation, and a comprehensive and wide-embracing spirit of reflection, are far too troublesome, obscure, and unproductive to have any attractions for their minds.

An unprejudiced clear perception, an indestructible repose, great perseverance, and a habit of discussing every subject with perfect freedom, are admirable guides to them along their favourite path, which is the more readily pursued, as they seldom permit themselves to be diverted by extraneous circumstances. The English are certainly less influenced by the opinions of others than any other people that I know.

From their quiet observing manner, which is almost always combined with a most laudable love of truth, they escape many of the quicksands and rocks, on which others, who indulge more in speculation and phantasies, make a shipwreck. And when they occasionally elevate themselves to subjects of higher speculation, their national self-confidence is often of the greatest use; hence many of the most valuable physiological and pathological discoveries we owe to Englishmen. What influence have the discoveries of Hunter, Bell, and others had on numerous branches of medical science.

Their surgeons exhibit the same qualities as operators, as their physicians do in medical practice: quiet, self-confident, and determined, and often very bold. Neatness and dispatch are not much aimed at; but downright roughness I never saw except in one, who is however considered as perhaps the most celebrated for his speed and dexterity. *Key, Liston, Tyrrell, Lawrence, Green, Travers,* and *Brodie* are the most distinguished; but I cannot suppress the remark that I saw one of these gentlemen perform an amputation of the thigh more badly than I could have believed.

After slowly making a circular incision through the integuments with two strokes of a large amputating knife, he began to dissect back the skin with its point, for about two inches. How long this took, any one may judge. The muscles were now divided down to the bone, and this step was repeated a second time; and then the bone was sawed across not sufficiently high up. At length, after upwards of five minutes suffering, a stump was formed with a projecting bone, a want of flesh and a superabundance of skin. Had such an occurrence taken place in any of the Paris hospitals the students would assuredly have hissed the surgeon out of the theatre."

The worthy doctor writes in the most grateful terms of the warm friendly reception he met with from his professional brethren in London, and closes his sketch of our medical institutions with an affectionate tribute to the talents of Sir Astley Cooper;—" who, although nearly seventy years of age, is still a very handsome man. His tall stout figure, beautiful head, and intelligent, lively and cheerful countenance, must at once strike the beholder. The large fortune that he has accumulated enables him to follow out his favourite anatomical pursuits with every advantage. He is not, like most successful professional men, still unsatisfied with his success; for, instead of hunting after some new mammon, we find him devoting his time, talents, and fortune to the advancement of professional science. At present he is engaged in an extensive work on the diseases of the mamma. His museum of preparations of the testicle and thymus gland, is unrivalled. It is to him that we owe the curious discovery that the latter of these organs serves to secrete during fœtal life a whitish fluid, resembling milk or chyle, which is conveyed by an excretory duct into the jugular vein, near the opening of the thoracic duct. This function seems to terminate soon after the birth of the child."

After visiting the leading provincial towns, Dr. *Varrentrapp* passed over to Dublin. On landing, he was at once struck with the wretched and filthy condition of the lower orders, " which is not altogether attributable to their mere poverty. A clean-combed head of hair on the poorest woman in Germany looks much better than the tawdry bonnet we see on the beggars here."

We will not follow our author in his description of the various hospitals, as the Richmond, the Whitworth, the Hardwicke Fever, Steevens', Mercers', &c., but at once come to his remarks on the Meath Hospital and medical officers, Drs. *Graves* and *Stokes*. He gives a very minute account of the opinions of the latter of these distinguished physicians on the nature and treatment of typhus fever as it occurs in Ireland. We shall give a brief extract.

"According to Stokes, typhus is an *essential* fever, which affects in an especial manner in different cases, and during different epidemics, either the head, the chest, or the abdomen. The ulcerations of the intestines, so much insisted upon by French pathologists, are neither of constant occurrence, nor are they characteristic of the disease; in one epidemic they are found on dissection much more frequently than in another. The character of the fever is usually inflammatory, but often putrid; petechiæ or miliary papulæ are very generally present; but they have no essential influence on the progress of the disease, nor can their presence affect our prognosis."

Our author here mentions a minutia in examining the alvine evacuations, which is certainly quite novel to us, and worthy indeed of the zeal of a pains-taking Deutscher: "not much advantage can be obtained from the mere inspection of the stools in a pôt-de-chambre; they ought to be shaken about in a glass vessel some hours before the visit of the physician, and the vessels should be left every morning at the bed-side of each patient!"

"The omission of this point," adds our author, "by *Stokes* is the more remarkable, as in every other respect he is a most assiduous and exact observer. His excellent work on the Diseases of the Chest, known to all the medical world, is a proof of this." * * * "The treatment recommended by *Stokes* is very different from that which is usually followed in Germany. The greater number of my countrymen have recourse to antiphlogistic measures, and to the use of clysters, fomentations to the abdomen, and cold lotions to the head; but a considerable number of the Irish and Scotch physicians pursue a very different plan. Occasionally indeed, but seldom, leeches are applied to the head when there are symptoms of congestion there, and to the abdomen when much pain is experienced. But it is chiefly when the thoracic symptoms are more than usually troublesome that local depletions are resorted to; and then the cupping-glasses are usually preferred to leeches. The application of cold to the head also is frequently employed. * * * The principal treatment, however, consists in the exhibition of cordials and stimulants whenever, and sometimes even before, the vital powers begin to sink.* Of such remedies wine, and especially Port wine, is by far the best. Brandy, and occasionally also small doses of spirit of turpentine, may be given with advantage at the same time in certain cases. When this mode of treatment is appropriate and is judiciously administered, the pulse almost invariably becomes less frequent, and at the same time more equable and full. Musk and camphor, in large doses, are sometimes of great utility."

Dr. *Varrentrapp*, who seems to have had very extensive practice in fever as it usually exhibits itself at Francfort, confesses that he is by no means a

---

* Dr. Stokes, it is well known, has directed the attention of physicians, in an especial manner, to the auscultatory phenomena of the heart as affording the most valuable signs to direct our treatment in typhus fever. Whenever the impulse of the beats is very feeble, and the sounds are faint and indistinct, we need have little fear of employing stimulants, whatever symptoms are present.

convert to the stimulating plan of treatment; although he admits that he saw himself several patients, in whom symptoms of congestion were very prominent, bear the use of wine much better than he could have believed. He adds :—

" Dr. *Graves* is much more cautious and temperate in the administration of wine than his colleague ; his practice is much more symptomatic, perhaps somewhat too much so. Other physicians in Dublin, especially some of the older practitioners, are very sceptical of the advantages of the wine-treatment in typhus."

In taking his leave of the Meath Hospital, Dr. *Varrentrapp* pronounces a high and well deserved eulogium on the talents of its two physicians, Drs. *Graves* and *Stokes.*

" The clinique of this hospital is certainly the very best that I have seen either in England or Ireland. Nowhere have I noticed so careful and patiently minute an examination of every symptom, so extended and so useful prelections on the diagnosis, and so regular a daily visit, (even on Sunday,) as here. The relation of the teacher to the pupils too is most generous, friendly, and truly inspiriting. The two ornaments of the Meath Hospital (Drs. *Graves* and *Stokes*), by their professional talents as well as by their courtesy, cannot fail to render this institution especially attractive to strangers."

The House of Recovery and Fever Hospital in Cork-street, seems to have occupied a good deal of the attention of our author. He gives an abstract of the views of Dr. O'Brien the senior physician, as explained in his late Treatise on Typhus.

" He has arranged the various forms of the disease under four heads;—1. When the brain and nervous system are chiefly affected—the *adynamic* and *ataxic* fever of French writers.—2. When the lungs chiefly suffer—the inflammatory or synochal form.—3. When the stomach and intestinal canal chiefly suffer—the gastric fever of *Pinel, Cheyne,* &c. the gastro-enterite of *Broussais ;* and—4. When the joints are more than usually affected—the rheumatic typhus of some authors.

The *first* of these forms is much the most dangerous, and in certain epidemics by far the most frequent too. The use of emetics in the early stage, and subsequently of wine, camphor, ammonia, &c. is chiefly indicated. In the *second* form, cupping on some part of the chest, blisters, and the exhibition of tartar-emetic are the most salutary remedies. In the *third,* the application of leeches over the epigastrium, and the use of calomel with opium, of gentle aperients, and of enemata, are chiefly useful; and in the *last* form, Dover's powder, calomel, purgatives, leeches and blisters are."

On these views of Dr. O'Brien, our author appends the following remarks :—

" Without doubt this review of the opinions and method of treatment at the first fever hospital of Ireland, which contains nearly 500 patients, possesses considerable interest; but it must be confessed, at the same time, that it clearly shews how several distinct morbid states are blended and grouped together by the author under one denomination. It will be observed, for example, that no distinction, either in a diagnostic or curative point of view, is made between a strictly bilious fever attended with congestion in the head and tendency to delirium, and genuine abdominal typhus : at least the mode of treatment recommended for the *third* groupe or form of the disease is applicable to gastric and bilious fever, but not to genuine abdominal typhus. The physician allows himself to attach too much consequence to the word '*fever.*' "

The worthy doctor seems to have left Dublin with the greatest regret, and mentions his great satisfaction at having met with such men as Drs. *Graves* and *Stokes*, Mr. *Porter* and others. He subsequently visited Scotland; but his stay in Edinburgh and Glasgow was so limited that he did little more than merely inspect the chief *lions* of these cities. He returned to London, where he spent a few days again, and thence crossed over to Belgium.

"At length, after the undisturbed enjoyment of so much that was beautiful and interesting in foreign lands, I returned, on a bright Summer evening in July, through rich and fertile plains to my father town, and joyful indeed was the end of my hundred-days' journey."

